# Supplementary material for: Viral shedding and symptom severity across populations during acute COVID in the ACTIV-2 study
Source: medRxiv. 2026 Feb 3:2026.01.31.26345293. Preprint. [Version 1] doi: 10.64898/2026.01.31.26345293 (PMC12889752; doi:10.64898/2026.01.31.26345293)
Supplement: 1 [file NIHPP2026.01.31.26345293V1-supplement-1.pdf]

medRxiv preprint doi: https://doi.org/10.64898/2026.01.31.26345293; this version posted February 3, 2026. The copyright holder for this preprint (which was not certified by peer review) is the author/funder, who has granted medRxiv a license to display the preprint in perpetuity.
It is made available under a CC-BY 4.0 International license .

Variable	β	95% CI	p-value
AGE	0.00	-0.02, 0.03	>0.9
SEX - F	—	—	—
SEX - M	-0.92	-1.5, -0.30	0.004
Race - BLACK	—	—	—
Race - HISPANIC	-0.60	-2.2, 1.0	0.5
Race - OTHER	1.2	-0.55, 2.9	0.2
Race - WHITE	-0.06	-1.6, 1.7	>0.9
Country/Region - North America	—	—	—
Country/Region - South Africa	-0.57	-2.3, 1.2	0.5
Country/Region - South America	0.35	-0.96, 1.7	0.6

Abbreviations: CI = Confidence Interval, β coefficient with 95% Confidence Interval

Supplemental Table 1. Multivariate analysis of SARS-CoV-2 viral load at study entry in those with quantifiable results and <=3 days from symptom onset.

medRxiv preprint doi: https://doi.org/10.64898/2026.01.31.26345293; this version posted February 3, 2026. The copyright holder for this preprint (which was not certified by peer review) is the author/funder, who has granted medRxiv a license to display the preprint in perpetuity.
It is made available under a CC-BY 4.0 International license .

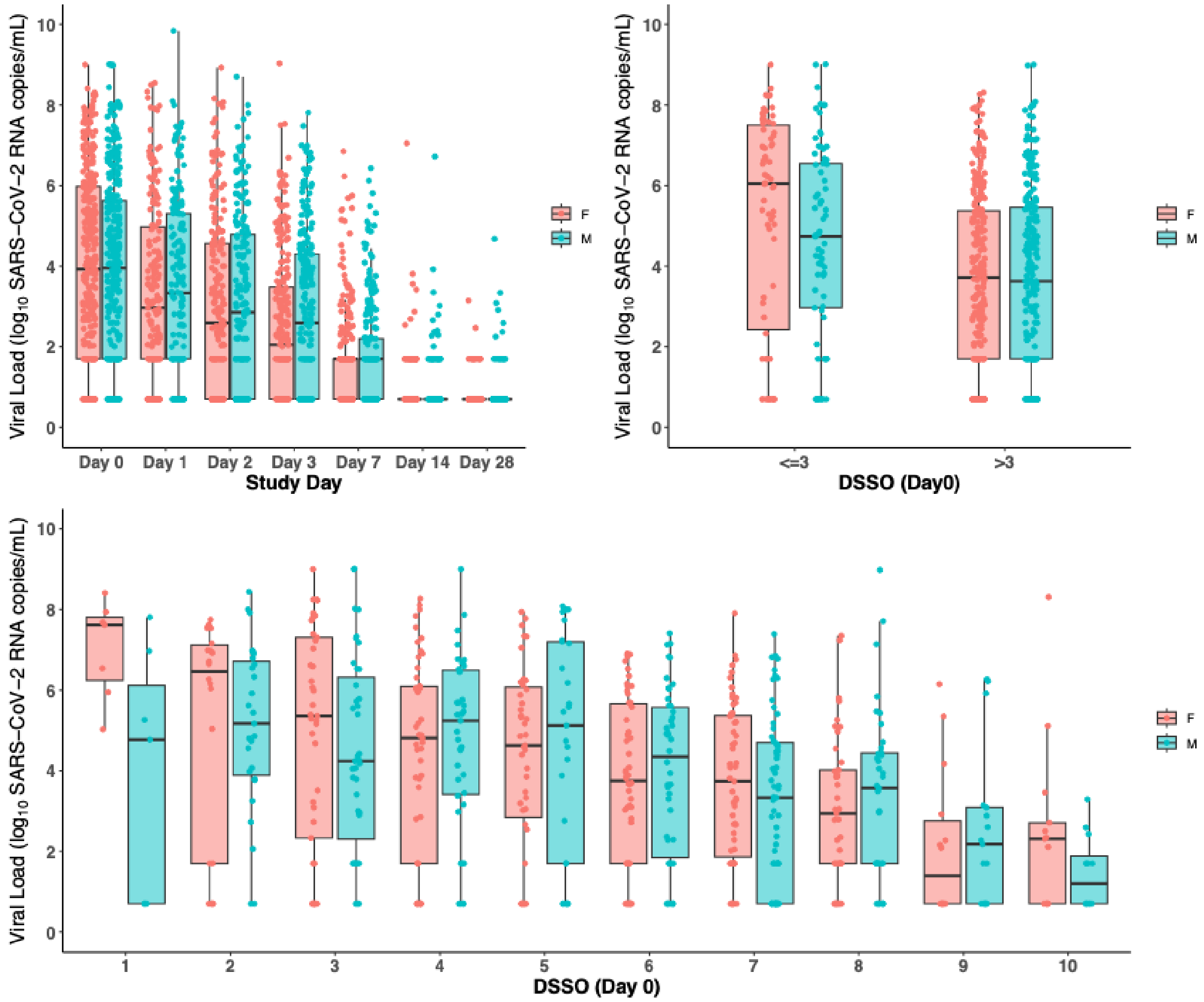

Supplementary Figure 1. Nasal SARS-CoV-2 viral load (VL) comparisons between males and females for 668 participants, including those with undetectable viral RNA levels at time of study entry. (A) VL by study day. (B) VL at enrollment (Day 0) stratified by ≤3 vs >3 days since symptom onset (DSSO). (C) VL at enrollment (Day 0) further categorized by individual DSSO days (1–10). Boxplots represent the interquartile range (IQR; 25th–75th percentile), with the horizontal line indicating the median. Individual data points are overlaid as dots. *P*-values were calculated using Wilcoxon rank-sum tests.

medRxiv preprint doi: https://doi.org/10.64898/2026.01.31.26345293; this version posted February 3, 2026. The copyright holder for this preprint (which was not certified by peer review) is the author/funder, who has granted medRxiv a license to display the preprint in perpetuity.
It is made available under a CC-BY 4.0 International license .

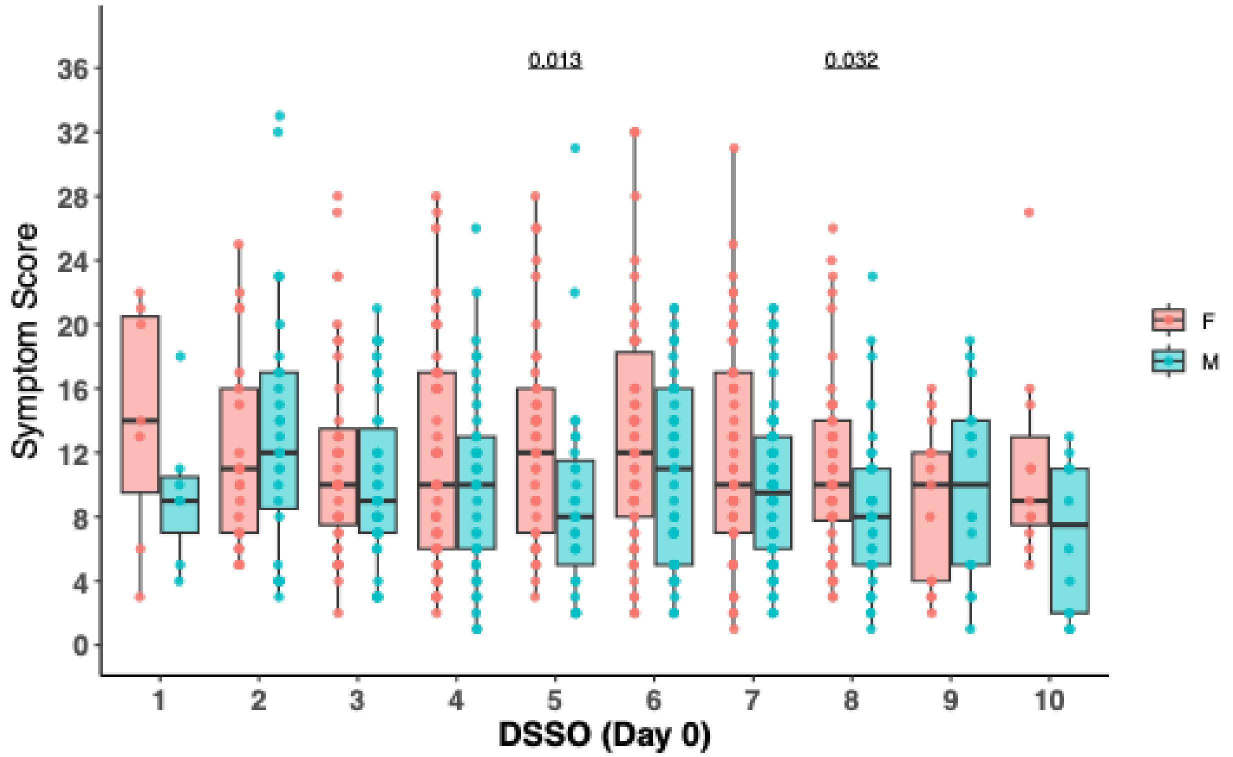

Supplemental Figure 2. Comparison of male and female symptom scores by days since symptom onset (DSSO) from secondary analysis including all participants. Data are shown as boxplots representing the interquartile range (IQR; 25th to 75th percentile) with the median as a horizontal line within the box. Individual data points are shown as dots. P-values were calculated using Wilcoxon rank-sum tests.
